# Burden of multiple high-risk factors in pregnancy before and after the universal two-child policy in Chinese women: An observational study

**DOI:** 10.7189/jogh.14.04134

**Published:** 2024-07-19

**Authors:** Yue Zhang, Weijie Ding, Xiaochen Dai, Hui Wang, Yangyang Cheng, Jiyue Dai, Xiaoqin Zhu, Xiaolin Xu

**Affiliations:** 1 School of Public Health, The Second Affiliated Hospital, Zhejiang University School of Medicine, Hangzhou, Zhejiang Province, China; 2 Health Care Department, Huai’an Maternal and Child Health Care Hospital Affiliated to Yangzhou University, Huai’an City, Jiangsu Province, China; 3 Department of Health Metrics Science, School of Medicine, University of Washington, Seattle, Washington, USA; 4 Institute for Health Metrics and Evaluation, University of Washington, Seattle, Washington, USA; 5 School of Public Health, Faculty of Medicine, The University of Queensland, Brisbane, Australia

## Abstract

**Background:**

The prevalence of high-risk pregnancy increased after the implementation of two-child policy in China, but the impact of this policy change on the burden and profile of multiple high-risk factors in pregnancy (MHFP) has been insufficiently explored. We hypothesised that the profile of MHFP might have changed after the two-child policy was implemented and aimed to estimate the prevalence, intercorrelation, and outcomes of MHFP before and after its introduction.

**Methods:**

We obtained data on the population of pregnant women before (2015) and after (2020/2021) the implementation of universal two-child policy in Huai’an. We then included 33 risk factors in our analysis based on the Five-Colour Management framework and defined MHFP as an individual having two or more of these factors. We also estimated the changes of the prevalence of each single factor and their coexistence. Lastly, we performed a network analysis to assess the intercorrelations across these factors and used logistic regression models to evaluate MHFP-related pregnancy outcomes.

**Results:**

We observed an increase in the prevalence of MHFP after the implementation of the universal two-child policy (25.8% in 2015 vs 38.4% in 2020/2021, *P* < 0.01). Chronic conditions (e.g. gestational diabetes mellitus, abnormal body mass index) had the largest increase among the included factors, while cardiovascular disease and hypertensive disorders were central factors of the network structures. The correlations of advanced maternal age with abnormal pregnancy histories and scarred uteri increased significantly from 2015 to 2020/2021. MHFP was associated with multiple pregnancy outcomes, including preterm birth (adjusted odds ratio (aOR) = 2.57; 95% confidence interval (CI) = 2.39–2.75), low birthweight (aOR = 2.77; 95% CI = 2.54–3.02), low Apgar score (aOR = 1.41; 95% CI = 1.19–1.67), perinatal death (aOR = 1.75; 95% CI = 1.44–2.12), and neonatal death (aOR = 1.76; 95% CI = 1.42–2.18). Moreover, an increasing number and certain combinations of MHFP were associated with higher odds of pregnancy outcomes. For example, the aOR of preterm birth increased from 1.67 (95% CI = 1.52–1.87) for one risk factor to 8.03 (95% CI = 6.99–9.22) for ≥4 risk factors.

**Conclusions:**

Chinese women experienced a higher burden of multiple high-risk factors after the introduction of the two-child policy, particularly those with advanced maternal age, obesity, and chronic conditions. Strategies targeting chronic conditions for women with MHFP should be prioritised and a shift to a multiple-factor-oriented framework is needed in the expanding Chinese maternal health care system.

A high-risk pregnancy (HRP) refers to the presence of risk factors such as coexisting health issues, advanced maternal age, and pregnancy complications, that makes the mother, the fetus, or the newborn baby at higher risks of adverse health outcomes [1,2]. There was an estimated 20 million women with HRPs worldwide in 2020, which are estimated to lead to over 800 maternal deaths every day [3–5]. The Chinese government has proposed the ‘universal two child policy’ in October 2015, which lead to an increased rate of women with multiparous births and advanced age [6]. Following this policy change, the National Health Commission of China proposed the Five-Colour Management framework for pregnant women in 2017 to better screen, assess, classify, and manage HRPs [7,8]. According to this framework, the estimated prevalence of HRPs in China ranged from 54.5% to 65.0% in 2019 [7]. The upward trend in prevalence suggested that China bears a higher burden of HRP, but the changes of the broad profile of these high-risk factors after the policy implementation remains unknown.

Recent studies have improved our understanding of some specific factors in pregnancy and their related outcomes, such as gestational diabetes mellitus (GDM) and hypertensive disorders [9–12], with recent research attention being diverted towards the coexistence of multiple high-risk factors in pregnancy (MHFP) [13–18]. For example, a cross-sectional study among the UK population estimated that the prevalence of coexisting multiple chronic conditions ranged from 19.8% to 46.2% in pregnant women and was associated with higher odds of preterm birth (adjusted odds ratio (aOR) = 1.64; 95% confidence interval (CI) = 1.48–1.82) [13,14]. Similarly, maternal multimorbidity was found to associate with preterm birth, low birthweight, and small for gestational age according to a study conducted in Japan [16]. Another study from India expanded the spectrum of MHFP by including maternal risks, lifestyle risks, medical risks, current health risks, and previous birth outcome risks, and found that 16.4% of the women in the study population had two or more high-risk factors simultaneously [18]. However, previous studies included different factors for MHFP measurement, making the comparison of MHFP prevalence and related causes and outcomes across different regions difficult. Moreover, while existing studies have reported on the prevalence of HRP or several certain risk factors in different areas of China, the exact prevalence of MHFP is not known. Considering that current vertical strategies focus mainly on single specific risk factor during pregnancy, investigating combinations of MHFPs, as well as MHFP-related causes and outcomes, could provide evidence for policy planning, particularly in terms of complex profiles of HRP following the child policy change.

We hypothesised that the burden and profile of MHFP might change following the implementation of two-child policy. Based on a population of pregnant women in Huai’an, a city in China, we aimed to assess the burden of MHFP and its coexisting combinations and networks before and after the introduction of the two-child policy, and to provide estimates for the associations of MHFP with pregnancy outcomes.

## METHODS

### Study population and data collection

We included pregnant women who had registered at maternity information system (MIS) in 2015 and 2020/2021 in Huai’an, China. The MIS is a regionally established maternal and child information system which hosts information from 58 health care centres of level I–III hospitals (31 in townships, 18 in counties, and 9 in municipalities) in Huai’an. The recorded information includes maternal characteristics; maternal health status before, during, and after pregnancy; pregnancy outcomes; neonatal and anthropometric characteristics; and neonatal health outcomes. Gynaecologists diagnosed diseases and anthropometric examinations, and also collected data on maternal characteristics and disease history through face-to-face interview when women were admitted. In our study, we enrolled pregnant women who gave birth between 1 January 2015 and 31 December 2015 or 1 July 2020 and 30 June 2021 in the samples for 2015 and 2020/2021, respectively. After cleaning, the data set contained data on 57 495 mothers in 2015 and 27 923 mothers in 2020/2021 (Figure S1 in the **Online Supplementary Document**).

### Assessment of high-risk factors in pregnancy

Information on high-risk factors was collected according to the Jiangsu Maternal Healthcare manual (2012 edition) in 2015 [19] and the updated National Health Commissions of the People’s Republic of China (2017 edition) in 2020/2021 [20]. We selected the specific high-risk factors in pregnancy for our study based on prior research [18] and the Five Colour Management framework, which is a case-by-case management framework classifying pregnant women into five colours levels (green, yellow, orange, red, and purple) according to their basic conditions and pregnancy complications [21]. The ‘yellow and above’ levels were considered as denoting HRPs. Per this framework, we included a subset of 33 risk factors for which data were available and grouped them into six categories: basic characteristics; pregnancy history; diseases history in gynaecology and obstetrics; pregnancy comorbidities; pregnancy complications; and infectious diseases (Table S1 in the **Online Supplementary Document**). We defined MHFP as the coexistence of two or more of these risk factors.

### Assessment of pregnancy outcomes

The pregnancy outcomes of interest were preterm birth, low birth weight/high birth weight, low score of five-minute Apgar, perinatal death, and neonatal death. They were assessed by obstetrician at birth and extracted from MIS records. Preterm birth was defined as infants born alive between 24–37 completed weeks of gestation [22]. Birth weight was measured using a digital scale and was categorised into three groups of low birth weight (<2.5 kg), normal birth weight (2.5–4.0 kg), and high birth weight (>4.0 kg) [23,24]. Obstetrician also assessed the five-minute Apgar scores, whereby a score <7 was considered low [25]. Perinatal death was defined as the death of foetus between 28 weeks of gestation and seven days after birth; neonatal death was defined as death within the first 28 days after delivery [26].

### Assessment of covariates

We also collected covariates such as hospital levels, maternal education levels, maternal employment status, gravidity and parity before current pregnancy. We retrieved data on hospital levels from the MIS. Maternal education levels (middle school and below, high school or technical secondary school, junior college and above), employment status (unemployed, employed or self-employed, and others), gravidity (0, 1, 2, ≥3) and parity (0, 1, ≥2) were obtained through face-to-face interview by gynaecologists. We also assessed the socioeconomic status (SES) of participants using the summed score (0, 1, or 2) of education level (0: high school and below; 1: college and above) and employment status (0: unemployed; 1: employed/self-employed/others), with a higher score indicating higher SES. We further classified the SES score into low (0) vs middle or high (1–2) levels.

### Statistical analysis

We summarised the basic characteristics of pregnant women as medians and interquartile ranges for continuous variables and as frequencies and percentages for categorical variables. We compared the variables characterised by MHFP status and collection years using Wilcoxon’s test or χ^2^ test.

Our aim was to assess the changes of MHFP and its combinations before (2015) and after (2020/2021) the implementation of universal two-child policy. To do this, we first calculated the prevalence of each single risk factor and estimated changes of the prevalence for each factor between 2015 and 2020/2021, after which we used UpSet plots to identify the most frequent combinations of these factors in 2015 and 2020/2021. We then conducted network analysis to assess the complex intercorrelations across these 33 risk factors, with risk factors being visualised as nodes and their interconnections being presented as edges across nodes. We constructed Ising models to estimate the network structure, which combined logistic regression with model selection to identify the relationships between risk factors [27]. This approach was appropriate for binary data. We then used the eLasso algorithm to confirm the best connection between each risk factor [27] and the extend Bayesian information criterion for model optimisation [28]. The penalty parameter game was set to 0.25 as recommended to obtain a sparse model [28]. Here we calculated the centrality indices to quantify the centrality of nodes in the network with metrics of strength, betweenness, and closeness. The first metric indicated directed connectivity, while the latter two denoted indirect connectivity. Among these, the strength metric was suggested to be a more reliable metric to estimate the central role of nodes [29,30]. We checked the accuracy of edge weights and stability of centrality indices using the bootstrapping method, which was conducted 1000 times [31]. Lastly, we performed the network comparison test to evaluate the difference of network structure between the two models in 2015 and 2020/2021.

In view of MHFP-related pregnancy outcomes, we set up multivariable logistic regression models to assess the association of MHFP, number of risk factors, and different MHFP combinations with pregnancy outcomes by calculating ORs and 95% CIs, adjusted for hospital level, education level, employment status, gravidity, and parity. We used restricted cubic splines to estimate the dose-response relationship between number of risk factors and different pregnancy outcomes, with four knots at the 25th, 50th, 75th, and 95th percentiles, according to the smallest Bayesian information criterion and Akaike information criterion [32]. We additionally performed subgroup analyses to assess the associations between MHFP and pregnancy outcomes, stratified by socioeconomic factors, alongside a sensitivity analysis to assess the association of MHFP and number of risk factors with pregnancy outcomes by imputing missing covariates through multiple imputation using the multiple imputation procedure [33]. Here we imputed the missing variables five times by the fully conditional method, which is also known as chained equations [34].

We set our significance threshold at the 0.05 level, using two sided tests. We performed the statistical analyses either in SAS, version 9.4 (SAS Institute Inc., North Carolina, USA) or R, version 4.1.2 (R Core Team, Vienna, Austria) and its ‘IsingFit’ and ‘NetworkComparisonTest’ [35] packages.

## RESULTS

### Characteristics of participants

The prevalence of MHFP was 25.80% (n = 14 833) and 38.41% (n = 10 725) in 2015 and 2020/2021. Women with MHFP were more likely to be older; in grade III hospitals; unemployed; overweight, or obese; had higher gravidity and parity before current pregnancy, and tended to deliver children with preterm birth; low/high birth weight; low score of five-minute Apgar; perinatal death; and neonatal death, compared with women without MHFP in both 2015 and 2020/2021. Moreover, women with MHFP in 2015 tended to have lower education, while those in 2020/2021 tended to be higher educated (**Table 1**; Table S2 in the **Online Supplementary Document**).

**Table 1:** Basic characteristics of pregnant women by MHFP in Huai’an in 2015 and 2020/2021*

	2015				2020/2021			
	**Total**	**Women without MHFP**	**Women with MHFP**	***P*-value**	**Total**	**Women without MHFP**	**Women with MHFP**	***P*-value**
	57 495 (100)	42 662 (74.2)	14 833 (25.8)		27 923 (100)	17 198 (61.6)	10 725 (38.4)	
**Maternal age, median (interquartile range)**	26.0 (5.0)	26.0 (4.0)	28.0 (8.0)	<0.001	28.0 (6.0)	27.0 (5.0)	30.0 (6.0)	<0.001
**Hospital level**				<0.001				<0.001
Grade I	17 567 (30.6)	13 703 (32.1)	3864 (26.1)		2238 (8.0)	1588 (9.2)	650 (6.1)	
Grade II	23 028 (40.1)	17 280 (40.5)	5748 (38.8)		9341 (33.5)	5894 (34.3)	3447 (32.1)	
Grade III	16 900 (29.4)	11 679 (27.4)	5221 (35.2)		16 344 (58.5)	9716 (56.5)	6628 (61.8)	
**Education level**				0.001				<0.001
Middle school and below	35 419 (61.6)	26 117 (61.2)	9302 (62.7)		10 311 (36.9)	6426 (37.4)	3885 (36.2)	
High school or technical secondary school	10 628 (18.5)	7903 (18.5)	2725 (18.4)		7726 (27.7)	4513 (26.2)	3213 (30.0)	
Junior college and above	11 448 (19.9)	8642 (20.3)	2806 (18.9)		9802 (35.1)	6204 (36.1)	3598 (33.5)	
Missing	-	-	-		84 (0.3)	55 (0.3)	29 (0.3)	
**Employment status**				0.040				0.001
Unemployed	19 754 (34.4)	14 596 (34.2)	5158 (34.8)		609 (2.2)	328 (1.9)	281 (2.6)	
Employed or self-employed	33 335 (58.0)	24 850 (58.2)	8485 (57.2)		15 001 (53.7)	9226 (53.6)	5775 (53.8)	
Others	4406 (7.7)	3216 (7.5)	1190 (8.0)		12 222 (43.8)	7587 (44.1)	4635 (43.2)	
Missing	-	-	-		91 (0.3)	57 (0.3)	34 (0.3)	
**BMI before pregnancy**				<0.001				<0.001
Normal weight	40 587 (70.6)	33 170 (77.8)	7417 (50.0)		17 683 (63.3)	12 988 (75.5)	4695 (43.8)	
Underweight	4618 (8.0)	2150 (5.0)	2468 (16.6)		1794 (6.4)	821 (4.8)	973 (9.1)	
Overweight	11 124 (19.3)	6893 (16.2)	4231 (28.5)		6465 (23.2)	2925 (17.0)	3540 (33.0)	
Obese	1166 (2.0)	449 (1.1)	717 (4.8)		1981 (7.1)	464 (2.7)	1517 (14.1)	
**Gravidity before current pregnancy**				<0.001				<0.001
0	23 296 (40.5)	19 113 (44.8)	4183 (28.2)		9301 (33.3)	6906 (40.2)	2395 (22.3)	
1	19 938 (34.7)	14 762 (34.6)	5176 (34.9)		8094 (29.0)	5205 (30.3)	2889 (26.9)	
2	8352 (14.5)	5429 (12.7)	2923 (19.7)		5570 (19.9)	3185 (18.5)	2385 (22.2)	
≥3	5909 (10.3)	3358 (7.9)	2551 (17.2)		4958 (17.8)	1902 (11.1)	3056 (28.5)	
**Parity before current pregnancy**				<0.001				<0.001
0	38 879 (67.6)	28 895 (67.7)	9984 (67.3)		13 113 (47.0)	9382 (54.6)	3731 (34.8)	
1	17 060 (29.7)	12 841 (30.1)	4219 (28.4)		12 482 (44.7)	6752 (39.3)	5730 (53.4)	
≥2	1556 (2.7)	926 (2.2)	630 (4.2)		2328 (8.3)	1064 (6.2)	1264 (11.8)	
**Preterm birth**	2158 (3.8)	1071 (2.5)	1087 (7.3)	<0.001	1683 (6.0)	649 (3.77)	1034 (9.6)	<0.001
**Low birth weight**	1518 (2.6)	779 (1.8)	139 (5.0)	<0.001	900 (3.2)	295 (1.7)	605 (5.6)	<0.001
**High birth weight**	5585 (9.7)	4007 (9.4)	1578 (10.6)	<0.001	2378 (8.5)	1342 (7.8)	1036 (9.7)	<0.001
**Low score of five-minute Apgar**	402 (0.7)	248 (0.6)	154 (1.0)	<0.001	201 (0.7)	108 (0.6)	93 (0.9)	0.022
**Perinatal death**	287 (0.5)	167 (0.4)	120 (0.8)	<0.001	164 (0.6)	77 (0.5)	87 (0.8)	<0.001
**Neonatal death**	332 (0.6)	195 (0.5)	137 (0.9)	<0.001	38 (0.1)	11 (0.1)	27 (0.3)	<0.001

BMI – body mass index, MHFP – multiple high-risk factors in pregnancy

*Presented as n (%) unless specified otherwise.

### Changes of the prevalence for single high-risk factor

**Figure 1:**
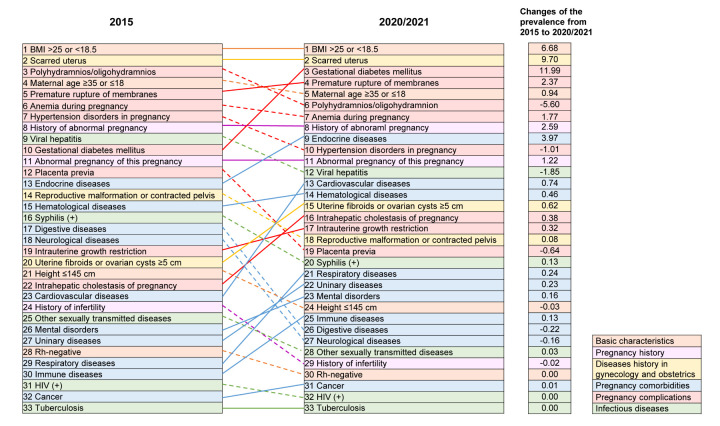
Ranks and changes of the prevalence of high-risk factors in pregnancy in 2015 and 2020/2021.

The top five most prevalent risk factors in 2015 were an abnormal body mass index (BMI) (>25 or <18.5) (20.29%), scarred uteri (17.20%), polyhydramnios or oligohydramnios (14.94%), maternal age ≥35 or ≤18 (10.00%), premature ruptures of membranes (PROM) (8.95%). They were likewise the top five risk factory by prevalence in 2020/2021 (abnormal BMI: 26.97%, scarred uteri: 26.90%, GDM: 13.54%, PROM: 11.32%, maternal age ≥35 or ≤18: 10.94%). We observed the largest increase in prevalence from 2015 to 2020/2021 for GDM, scarred uteri, and abnormal BMIs (more than 6% increase), while polyhydramnios or oligohydramnios, viral hepatitis, and hypertension disorders in pregnancy (HDP) had the largest declines. In view of factors, comorbid chronic conditions saw the greatest increases, while infectious diseases mainly decreased (**Figure 1**).

### Combinations of high-risk factors

**Figure 2:**
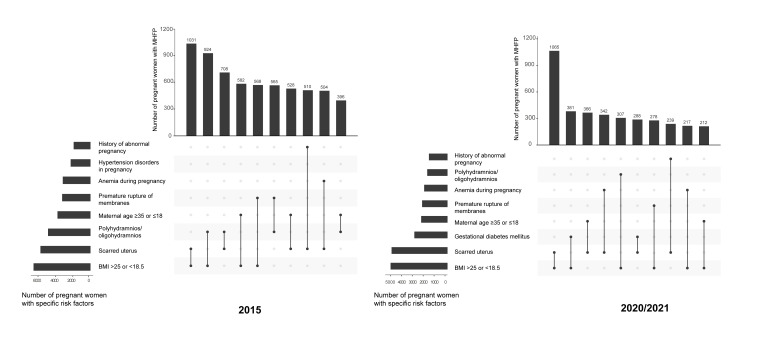
Ranking of the first ten leading combinations of high-risk factors in pregnancy in 2015 and 2020/2021.

The five most frequent combinations of MHFP in 2015 were the coexistence of an abnormal BMI and a scarred uterus; an abnormal BMI and polyhydramnios or oligohydramnios; a scarred uterus and polyhydramnios or oligohydramnios; an abnormal BMI and age ≥35 or ≤18; and polyhydramnios or oligohydramnios and PROM. Similarly, the most frequent combination in 2020/2021 was also the coexistence of an abnormal BMI and a scarred uterus, followed by the coexistence of an abnormal BMI and GDM, a scarred uterus and age ≥35 or ≤18, a scarred uterus and anaemia, and an abnormal BMI and polyhydramnios or oligohydramnios (**Figure 2**).

### Network structure of MHFP

**Figure 3:**
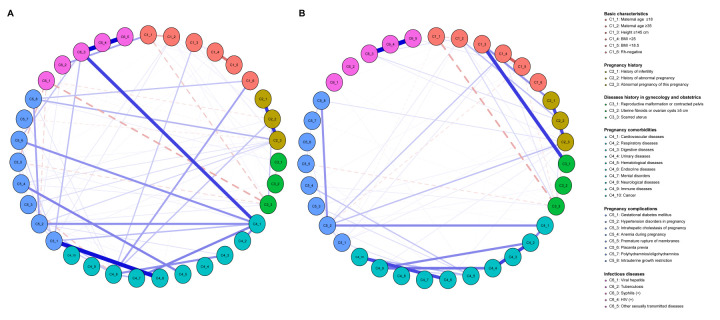
Network structure of MHFP in 2015 and 2020/2021. **Panel A.** The graph depicts the analysis network of MHFP in 2015. **Panel B.** The graph depicts the analysis network of MHFP in 2020-21. The nodes represent each high-risk factor in the model, and the edges connecting the nodes represent the effect size for the association between nodes. Blue and red edges represent positive and negative connections, respectively. The colours of the nodes correspond to the groups of each factor.

Regarding the network structures of MHFP in 2015 and 2020/2021, the analysis of the centrality measures of nodes showed that cardiovascular disease and neurological diseases were the central factors with the greatest connection on the overall structure of the network in 2015, while HDP and respiratory disease were the central factors in 2020/2021. The stability and accuracy of the network structure performed well in this study. Specifically, the comparison of network in 2015 and 2020/2021 showed no significant global difference (global strength: 2015 = 82.28, 2020/2021 = 61.52; *P* = 0.78). The edge weight between ‘syphilis’ and ‘sexually transmitted diseases’ were the highest in both networks. Nevertheless, there were several significant differences among the edges; for example, we saw significant increases (*P* < 0.05) in the correlations of maternal age ≥35 and history of abnormal pregnancy; maternal age ≥35 and scarred uteri; and a BMI>25 and GDM (**Figure 3**; Figures S3–4 and Supplementary Data S1–2 in the **Online Supplementary Document**).

### Association of MHFP and number of risk factors with pregnancy outcomes

**Figure 4:**
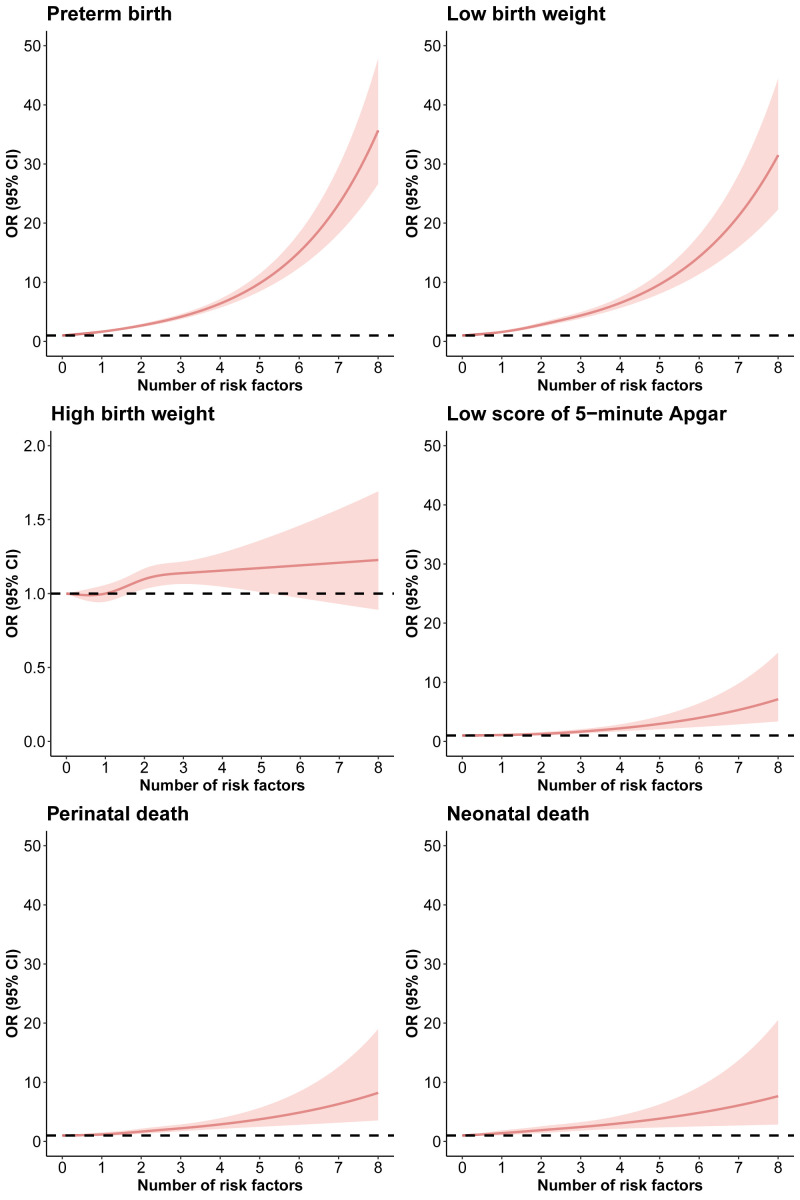
Restricted cubic spline models of the association between number of risk factors and pregnancy outcomes in the total population. Models were adjusted for hospital level, education level, employment status, gravidity, and parity.

MHFP was associated with higher odds of multiple pregnancy outcomes, including preterm birth (aOR = 2.57; 95% CI = 2.39–2.75), low birth weight (aOR = 2.77; 95% CI = 2.54–3.02), high birth weight (aOR = 1.14; 95% CI = 1.09–1.20), low score of five-minute Apgar (aOR = 1.41; 95% CI = 1.19–1.67), perinatal death (aOR = 1.75; 95% CI = 1.44–2.12), and neonatal death (aOR = 1.76; 95% CI = 1.42–2.18). Moreover, the odds of adverse pregnancy outcomes tend to increase with the rise number of risk factors (**Table 2**). For example, the OR of preterm birth ranged from 1.67 (95% CI = 1.52–1.87) for one risk factor to 8.03 (95% CI = 6.99–9.22) for ≥4 risk factors. The restricted cubic spline models also showed a dose-response relationship between number of risk factors and most adverse pregnancy outcomes (**Figure 4**). The subgroup analyses suggested that the associations between MHFP and almost all adverse pregnancy outcomes (except for high birth weight) were more evident among women with low SES, while the sensitivity analysis showed similar results to the main findings (Figure S5 and Table S3 in the **Online Supplementary Document**).

**Table 2:** Association of MHFP and number of risk factors with pregnancy outcomes*

	Total population		2015		2020/2021	
**MHFP vs non-MHFP**	**n (%)**	**aOR (95% CI)**	**n (%)**	**aOR (95% CI)**	**n (%)**	**aOR (95% CI)**
Preterm birth	2121 (8.3)	2.57 (2.39–2.75)	1087 (7.3)	2.75 (2.51–3.02)	1034 (9.6)	2.62 (2.36–3.91)
Low birth weight	1344 (5.3)	2.77 (2.54–3.02)	139 (5.0)	2.58 (2.32–2.88)	605 (5.6)	3.61 (3.11–4.18)
High birth weight	2614 (10.2)	1.14 (1.09–1.20)	1578 (10.6)	1.10 (1.03–1.17)	1036 (9.7)	1.24 (1.14–1.36)
Low score of 5-min Apgar	247 (1.0)	1.41 (1.19–1.67)	154 (1.0)	1.61 (1.31–1.99)	93 (0.9)	1.36 (1.01–1.82)
Perinatal death	207 (0.8)	1.75 (1.44–2.12)	120 (0.8)	1.85 (1.45–2.36)	87 (0.8)	1.95 (1.42–2.69)
Neonatal death	164 (0.6)	1.76 (1.42–2.18)	137 (0.9)	1.80 (1.43–2.26)	27 (0.3)	4.36 (2.11–8.99)
**Number of risk factors**						
Preterm birth						
*0*	574 (2.1)	ref	341 (1.7)	ref	233 (3.1)	ref
*1*	1146 (3.6)	1.69 (1.52–1.87)	730 (3.3)	1.97 (1.73–2.25)	416 (4.3)	1.41 (1.20–1.66)
*2*	972 (5.8)	2.62 (2.35–2.91)	568 (5.4)	3.19 (2.77–3.67)	404 (6.4)	2.20 (1.86–2.60)
*3*	658 (10.6)	4.52 (4.00–5.10)	327 (10.1)	5.69 (4.82–6.72)	331 (11.2)	3.97 (3.31–4.75)
*≥4*	491 (12.8)	8.03 (6.99–9.22)	192 (18.1)	9.54 (7.73–11.78)	299 (20.0)	7.60 (6.25–9.23)
Low birth weight						
*0*	372 (1.3)	ref	271 (1.3)	ref	101 (1.4)	ref
*1*	702 (2.2)	1.64 (1.44–1.86)	508 (2.3)	1.74 (1.50–2.02)	194 (2.0)	1.58 (1.23–2.01)
*2*	632 (3.8)	2.80 (2.45–3.20)	390 (3.7)	2.79 (2.38–3.28)	242 (3.9)	3.30 (2.60–4.19)
*3*	406 (6.6)	4.73 (4.07–5.49)	210 (6.5)	4.61 (3.80–5.59)	196 (6.6)	5.98 (4.65–7.70)
*≥4*	306 (12.0)	8.48 (7.17–10.04)	139 (13.1)	8.59 (6.82–10.83)	167 (11.2)	10.69 (8.17–14.01)
High birth weight						
*0*	2471 (8.8)	ref	1905 (9.3)	ref	566 (7.6)	ref
*1*	2878 (9.1)	1.00 (0.95–1.06)	2101 (9.5)	0.99 (0.93–1.06)	776 (8.0)	1.02 (0.91–1.14)
*2*	1669 (9.9)	1.10 (1.03–1.17)	1097 (10.4)	1.06 (0.98–1.15)	572 (9.1)	1.17 (1.04–1.33)
*3*	673 (10.9)	1.24 (1.13–1.36)	366 (11.3)	1.16 (1.03–1.31)	307 (10.4)	1.37 (1.18–1.60)
*≥4*	272 (10.6)	1.28 (1.11–1.46)	115 (10.8)	1.17 (0.95–1.43)	157 (10.5)	1.44 (1.18–14.75)
Low score of five-minute Apgar						
*0*	155 (0.6)	ref	109 (0.5)	ref	46 (0.6)	ref
*1*	201 (0.6)	1.08 (0.87–1.33)	139 (0.6)	1.15 (0.89–1.48)	62 (0.6)	1.06 (0.72–1.56)
*2*	0.8 (0.8)	1.24 (0.98–1.58)	88 (0.8)	1.47 (1.11–1.97)	42 (0.7)	1.14 (0.74–1.75)
*3*	68 (1.1)	1.63 (1.21–2.19)	40 (1.2)	2.00 (1.37–2.90)	28 (1.0)	1.54 (0.94–2.51)
*≥4*	49 (1.9)	2.59 (1.84–3.64)	26 (2.4)	3.50 (2.23–5.49)	23 (1.5)	2.34 (1.37–4.00)
Perinatal death						
*0*	101 (0.4)	ref	67 (0.3)	ref	34 (0.5)	ref
*1*	143 (0.6)	1.18 (0.92–1.53)	100 (0.5)	1.34 (0.98–1.83)	43 (0.5)	1.04 (0.66–1.64)
*2*	106 (0.6)	1.59 (1.20–2.09)	67 (0.6)	1.82 (1.29–2.67)	39 (0.6)	1.57 (0.98–2.51)
*3*	64 (1.0)	2.40 (1.74–3.32)	34 (1.1)	2.72 (1.78–4.17)	30 (1.0)	2.54 (1.52–4.25)
*≥4*	37 (1.5)	3.07 (2.06–4.57)	19 (1.8)	3.99 (2.34–6.79)	18 (1.2)	2.95 (1.61–5.43)
Neonatal death						
*0*	78 (0.3)	ref	74 (0.4)	ref	4 (0.1)	ref
*1*	128 (0.4)	1.41 (1.06–1.87)	121 (0.6)	1.47 (1.09–1.96)	7 (0.1)	1.47 (0.43–5.06)
*2*	89 (0.5)	1.84 (1.35–2.51)	79 (0.8)	1.93 (1.40–2.67)	10 (0.2)	3.54 (1.09–11.48)
*3*	46 (0.7)	2.50 (1.72–3.65)	36 (1.1)	2.60 (1.73–3.92)	10 (0.3)	7.88 (2.39–26.01)
*≥4*	29 (1.1)	3.66 (2.34–5.74)	22 (2.1)	4.18 (2.54–6.88)	7 (0.5)	11.36 (3.13–41.20)

aOR – adjusted odds ratio, CI – confidence interval, MHFP – multiple high-risk factors in pregnancy, ref – reference

*Models were adjusted for hospital level, education level, employment status, gravidity, and parity.

### Association of MHFP combinations with pregnancy outcomes

Different MHFP combinations were associated with most of adverse pregnancy outcomes in both 2015 and 2020/2021 (Table S4 in the **Online Supplementary Document**). For the five most frequent combinations of MHFP, the coexistence of an abnormal BMI and PROM was associated with the highest odds of preterm birth (aOR = 8.39; 95% CI = 6.50–10.74), low birth weight (aOR = 4.77; 95% CI = 3.43–6.61), and low Apgar score (aOR = 2.43; 95% CI = 1.26–4.68) in 2015, while the coexistence of a scarred uterus and anaemia was associated with highest odds of preterm birth (aOR = 2.95; 95% CI = 2.21–3.94) and low birth weight (aOR = 3.89; 95% CI = 2.61–5.82) in 2020/2021. Specially, the coexistence of abnormal BMI and GDM was mostly associated with high birth weight of offspring (aOR = 2.35; 95% CI = 1.91–2.89).

## DISCUSSION

### Principle findings

Our results showed a higher burden of MHFP in China after two-child policy implementation, with the prevalence increasing from 25.8% in 2015 to 38.4% in 2020/2021. Among the factors, GDM, a scarred uterus, and abnormal BMI had the largest increase in prevalence. The top two most prevalent risk factors (both in 2015 and 2020/2021) were abnormal BMI and scarred uterus, and the most frequent combination was the coexistence of abnormal BMIs and a scarred uterus. The network analysis showed that cardiovascular and neurological diseases were the central factors with the greatest connection with other factors in 2015, while HDP and respiratory disease were the central factors in 2020/2021. The correlations of advanced maternal age with abnormal pregnancy history and a scarred uterus increased significantly from 2015 to 2020-21. Lastly, we found MHFP to be associated with higher risks of adverse pregnancy outcomes, including preterm birth, delivering children with low birth weight, a low Apgar score, perinatal death, and neonatal death.

### Comparison with previous literature

We found a high prevalence of pregnant women with at least one risk factor during pregnancy, which also increased from 64.18% in 2015 to 73.26% in 2020/2021. This aligns with a previous study reporting a HRP prevalence of 54.5–65.0% in China across four cities in 2019 [7], and is similar to findings from other settings such as Iran (75%) [36] and Korea (71.7%) [37]. However, this observed prevalence is higher than that of countries like India (49.4%) [18], Nigeria (21.5%) [38], and Ethiopia (26.4%) [39]. These variations may partly be explained by differences in the definitions and screening frameworks of HRP; diagnostic accuracy; the quality of antenatal, intranatal, and postnatal interventions; and socioeconomic differences across different regions.

More importantly, we observed that over a quarter of women with HRP had two or more risk factors simultaneously; this rate increased with the introduction of the two-child policy. This growing prevalence may be attributed to several causes. First, the higher burden of certain chronic conditions, such as obesity, hypertension, diabetes, and heart diseases, has increased the proportion of HRP women accompanied with related pregnancy comorbidities and complications [40]. Accordingly, a significant burden of multimorbidity among pregnancy women was found in several contexts, with an estimated prevalence of 10.2% in UK [14], 6.3% in Japan [16], and 2.3% in China [17]. Second, the easing of childbirth policy has directly led to the increased proportion of multiparous pregnancies [6,41]. As a result, more women gave birth at older ages after the policy change [41], contributing to the higher risk of age-related pregnancy complications, such as pre-eclampsia and GDM [42]. Third, as China has been facing a relatively high rate of caesarean deliveries [43], the rate of pregnant women with uterine scars has correspondingly doubled, which resulted in downstream adverse pregnancy outcomes [41,42].

In terms of individual risk factors, we observed a high increase in the prevalence of GDM, scarred uteri, and abnormal BMIs. The increase in the prevalence of GDM was also reported in other populations of China, such as the one observed in Ningbo (from 4% in 2010 to 21% in 2020) [44]. We further found that most comorbid chronic conditions during pregnancy had increased ranks of prevalence for each factor in 2015 and 2020/2021, while infectious diseases had decreased ranks. This aligns with the spectrum changes of disease burden in the general population and could possibly worsen in pregnant women with advanced maternal age or obesity [44–46]. These changes remind us that strategies targeting chronic conditions should be prioritised for women with MHFP.

Abnormal BMI and scarred uteri were the most frequent two risk factors in both 2015 and 2020/2021, while the coexistence of an abnormal BMI and a scarred uterus was the most prevalent combination in both years. The rising rate of underweight and overweight/obesity in Chinese women of reproductive age was also observed in a previous study [22], while the increase in caesarean delivery rates and the higher proportion of multiparas would contribute to the increasing prevalence of scarred uteri [47]. Regarding their close connection, maternal obesity was reported as an important predictor of caesarean delivery [41,48,49], thereby contributing to the high prevalence of the combination of abnormal BMI and scarred uteri in subsequent births. Managing obesity in women of reproductive age is a lengthy process, the monitoring of which may need to be continually intensified.

The network analysis identified cardiovascular and neurological diseases as the central factors with the greatest connection to other factors in 2015. According to the network structure, cardiovascular disease during pregnancy was positively correlated with seven factors: respiratory diseases; mental disorders; GDM; HDP; PROM; placenta previa; and syphilis. In line with a previous study, the rate of pregnant women who had some type of heart disease as a comorbid condition increased sharply in 2014, mainly due to the increasing number of women with congenital heart disease reaching reproductive age [50]. Neurological disease was also positively correlated with seven factors, suggesting a great burden of these diseases among pregnant women in 2015, such as epilepsy and hypophrenia, which were particularly prevalent in low- or middle-income areas in China [51,52]. In 2020/2021, HDP and respiratory disease were the central factors positively correlated with 14 and 3 other factors, respectively. The prevalence of HDP in China has increased significantly in recent years following the growing prevalence of obesity and advanced maternal age [53]. Our results further suggested that a strong association exists between HDP and other factors affecting maternal health, which should be the core of maternal care system. Furthermore, respiratory diseases were strongly associated with cardiovascular diseases, urinary diseases, and immune diseases, which are all chronic diseases comorbid with pregnancy. This aligns with the growing research interest in maternal multimorbidity that presents a significant health burden to women, as the disease profile in this population changes over time [13,14,54]. By identifying these core factors in MHFP, our study provides essential information for health care providers, highlighting which factors should be prioritised in the development of effective prevention and management strategies for pregnant women.

The comparison of MHFP network structure showed no significant global difference in 2015 and 2020/2021. However, we identified several differences across edges between the factors. We found that significant increases in the correlations of maternal age ≥35 and a history of abnormal pregnancy; maternal age ≥35 and scarred uteri; and BMIs >25 and GDM. These changes may be associated with the change in the childbirth policy in China signalling a trend of delayed childbearing age and higher rate of multipara women [6,55], which would further stress the need for expanded maternal health care for the increasing proportion of older maternal population.

We also found MHFP to be associated with multiple adverse pregnancy outcomes, including preterm birth, delivery of children with low/high birth weight, low Apgar scores, perinatal death, and neonatal death. More importantly, the risk of most adverse pregnancy outcomes would increase with the growing number of risk factors, suggesting the synergistic or multiplicative effects rather than the additive effects among these factors. Therefore, our findings emphasise the importance of measures to avert the progression from one risk factor to MHFP [15]. There is a need to balance the primary and secondary prevention against adverse pregnancy outcomes in pregnant women, especially in those who already have pre-pregnancy health problems.

### Strengths and limitations

To our knowledge, this is the first study to assess the prevalence of MHFP and MHFP-related pregnancy outcomes in China. One of its strengths is the assessment of the whole population of pregnant women in Huai’an, which amounted to a relatively large sample size. Furthermore, the usage of the network analysis approach allowed us to assess potential correlations across many risk factors and to identify critical factors in the network structure, which could facilitate further considerations of which factors to be prioritised in future research and policymaking. However, some limitations must be noted. First, we retrieved our data from the MIS, which has less information on disease severity and disease classification. For example, in view of mental health disorders, the MIS hosts data for schizophrenia and lacks exact assessments and records for other common conditions such as depression and anxiety. Also, due to the improvement in diseases diagnosis and screening technology, we might have underestimated the prevalence for some diseases in 2015. Second, MHFP is an emerging research area, which is why there is no standardised framework for identifying and categorising these factors. Although we have included 33 types of risk factors in the definition of MHFP based on the Five Colour Management, we might have still neglected some risk factors. These issues may undermine the validity of this study and make cross-study comparisons challenging. Systematic reviews and cohort studies are needed to create a criterion for the measurement and definition of MHFP taking different types of physical, mental, and social-behavioural high-risk factors into consideration [56]. Third, the cross-sectional study design only provided a snapshot of MHFP at two time points before and after the two-child policy was implemented, limiting the predictions about future trends of MHFP. Future studies with a longitudinal study design could provide more evidence on the relationship between this change in policy and MHFP, as well as the long-term health outcomes for children born to mothers with MHFP. Moreover, we included six types of adverse pregnancy outcomes to assess the influence of MHFP; therefore, some other outcomes should be considered in future analyses. Additionally, the second set of data we used was from 2020/2021, which is the period of the COVID-19 pandemic; this may have influenced the MHFP profile, as previous evidence suggested that the COVID-19 pandemic brought an increased prevalence of pregnancy comorbidities and complications [57]. However, the incidence of COVID-19 in Huai’an was low during the period of data collection, likely making the impact of pandemic on our results relatively small [58]. Finally, this is a regional survey which is not representative of the total Chinese population. Future studies would allow for comparisons of the geographical variations in MHFP prevalence within China, which could provide valuable insights into regional disparities and thereby inform intervention strategies targeted at different areas.

### Implications

Previous studies have explored the prevalence of certain risk factors in pregnancy and their associations with adverse outcomes, but few evaluated the whole risk factor profile and their coexistence. The novelty of our study includes the measurement of multiple factors simultaneously and the comparison of complex networks of MHFP before and after the two-child policy had been implemented, which is important for future considerations of which factors or combinations should be prioritised for policymaking and programme development. Our findings imply that there is a need to shift the focus of research from single risk factors to a broader spectrum of factors and identify their cross-associations. For example, Periyasamy and colleagues have conducted research on HRP considering a broad profile of multiple factors based on a population from India National Family Health Survey [18], while the MuM-PreDiCT Group has explored intervention strategies or programmes to improve maternity care for pregnant women with two or more health conditions [13,14,54]. Transnational and multidisciplinary studies could compare these findings with international data, which could provide a broader understanding of how policy changes and socioeconomic factors impact maternal health globally.

This study also has public health implications, particularly for maternal and child health care systems. The Chinese government has made great progress in maternal and child health care in the past few decades [59], but some new challenges emerged after the introduction of the two-child policy, particularly for advanced maternal age, scarred uteri, obesity, and chronic conditions, as we observed here as well. Corresponding strategies and measures should correspond to the perceived needs and the policy change. This recommendation can be implemented by paying special attention to women with advanced maternal age who also have a higher rate of caesarean delivery in previous pregnencies [6]; starting maternal health care before pregnancy, thereby complying with the increased burden of chronic conditions [14]; and shifting the vertically structured health care system towards horizontal frameworks beyond current HRP management based on individual risk factors. Notably, the high prevalence of HRP after the policy implementation (73.26% in this study) may cause increased risk of mental health disorders (such as stress and anxiety) [60]. Further studies to examine the potential impact of MHFP on maternal and postpartum mental health disorders are warranted.

## CONCLUSIONS

The burden of pregnancy with multiple high-risk factors has increased with the implementation of two-child policy in Huai’an, China. In particular, factors associated with advanced maternal age, obesity, and chronic conditions presented a significant health burden. The expanding Chinese maternal and child health care system should reflect and monitor the changes of risk factors profiles following the introduction of the new policy, and shift from a one-factor-focussed to a multiple-factor-oriented framework.

## Additional material


Online Supplementary Document

## References

[1] Soh MC Nelson-Piercy C High-risk pregnancy and the rheumatologist. Rheumatology (Oxford). 2015;54:572–87. 10.1093/rheumatology/keu394 25477056

[2] Sun L Yue H Sun B Han L Qi M Tian Z Estimation of birth population-based perinatal-neonatal mortality and preterm rate in China from a regional survey in 2010. J Matern Fetal Neonatal Med. 2013;26:1641–8. 10.3109/14767058.2013.794208 23570293 PMC3812698

[3] Holness N High-Risk Pregnancy. Nurs Clin North Am. 2018;53:241–51. 10.1016/j.cnur.2018.01.010 29779516

[4] Carvalheira AP Tonete VL Parada CM Feelings and perceptions of women in the pregnancy-puerperal cycle who survived severe maternal morbidity. Rev Lat Am Enfermagem. 2010;18:1187–94. 10.1590/S0104-11692010000600020 21340285

[5] World Health Organization. Maternal mortality. 2019. Available: https://www.who.int/news-room/fact-sheets/detail/maternal-mortality. Accessed: 2 November 2023.

[6] Li HT Xue M Hellerstein S Cai Y Gao Y Zhang Y Association of China’s universal two child policy with changes in births and birth related health factors: national, descriptive comparative study. BMJ. 2019;366:l4680. 10.1136/bmj.l4680 31434652 PMC6699592

[7] Liu Y Rong L Aiqun H The Distribution of Pregnant Women with Different Pregnancy Risks — 4 Cities, China, 2019. China CDC Wkly. 2021;3:50–3. 10.46234/ccdcw2021.016 34594955 PMC8392934

[8] Liu J Song L Qiu J Jing W Wang L Dai Y Reducing maternal mortality in China in the era of the two-child policy. BMJ Glob Health. 2020;5:e002157. 10.1136/bmjgh-2019-002157 32133196 PMC7042574

[9] Alejandro EU Mamerto TP Chung G Villavieja A Gaus NL Morgan E Gestational Diabetes Mellitus: A Harbinger of the Vicious Cycle of Diabetes. Int J Mol Sci. 2020;21:5003. 10.3390/ijms21145003 32679915 PMC7404253

[10] Koning SH Hoogenberg K Lutgers HL Van den Berg PP Wolffenbuttel BHR Gestational Diabetes Mellitus: current knowledge and unmet needs. J Diabetes. 2016;8:770–81. 10.1111/1753-0407.12422 27121958

[11] Wu P Green M Myers JE Hypertensive disorders of pregnancy. BMJ. 2023;381:e071653. 10.1136/bmj-2022-071653 37391211

[12] Metoki H Iwama N Hamada H Satoh M Murakami T Ishikuro M Hypertensive disorders of pregnancy: definition, management, and out-of-office blood pressure measurement. Hypertens Res. 2022;45:1298–309. 10.1038/s41440-022-00965-6 35726086 PMC9207424

[13] Lee SI Azcoaga-Lorenzo A Agrawal U Kennedy JI Fagbamigbe AF Hope H Epidemiology of pre-existing multimorbidity in pregnant women in the UK in 2018: a population-based cross-sectional study. BMC Pregnancy Childbirth. 2022;22:120. 10.1186/s12884-022-04442-3 35148719 PMC8840793

[14] Azcoaga-Lorenzo A Fagbamigbe AF Agrawal U Black M Usman M Lee SI Maternal multimorbidity and preterm birth in Scotland: an observational record-linkage study. BMC Med. 2023;21:352. 10.1186/s12916-023-03058-4 37697325 PMC10496247

[15] Tomlinson M O’Connor MJ le Roux IM Stewart J Mbewu N Harwood J Multiple risk factors during pregnancy in South Africa: the need for a horizontal approach to perinatal care. Prev Sci. 2014;15:277–82. 10.1007/s11121-013-0376-8 23475562 PMC3718865

[16] Nakanishi K Saijo Y Yoshioka E Sato Y Kato Y Nagaya K Association between maternal multimorbidity and preterm birth, low birth weight and small for gestational age: a prospective birth cohort study from the Japan Environment and Children’s Study. BMJ Open. 2023;13:e069281. 10.1136/bmjopen-2022-069281 36921942 PMC10030623

[17] Li J Li Y Duan Y Xiao X Luo J Luo M Dose-response associations of maternal age with pregnancy complications and multimorbidity among nulliparas and multiparas: A multicentric retrospective cohort study in southern China. J Glob Health. 2023;13:04117. 10.7189/jogh.13.04117 37767793 PMC10535007

[18] Kuppusamy P Prusty RK Kale DP High-risk pregnancy in India: Prevalence and contributing risk factors - a national survey-based analysis. J Glob Health. 2023;13:04116. 10.7189/jogh.13.04116 37712385 PMC10502764

[19] Municipal Health Bureau Huai’an Office. Notice on further strengthening the management of high-risk pregnant women. 2012. Available: http://wjw.huaian.gov.cn/col/6832_854731/art/sj_wjw_45471.html. Accessed: 21 October 2023.

[20] National Health Commission of the PRC. Overview: notice of the National Health and Family Planning Commission on strengthening the safety of mother and child. July 21, 2017. Available: http://www.nhc.gov.cn/fys/s3581/201711/9c3dc9b4a8494d9a94c02f890e5085b1.shtml. Accessed: 21 October 2023.

[21] Liu J Jing W Liu M Risk management of pregnant women and the associated low maternal mortality from 2008-2017 in China: a national longitude study. BMC Health Serv Res. 2022;22:335. 10.1186/s12913-022-07721-z 35287680 PMC8920427

[22] Blencowe H Cousens S Oestergaard MZ Chou D Moller AB Narwal R National, regional, and worldwide estimates of preterm birth rates in the year 2010 with time trends since 1990 for selected countries: a systematic analysis and implications. Lancet. 2012;379:2162–72. 10.1016/S0140-6736(12)60820-4 22682464

[23] Chudasama YV Khunti K Gillies CL Dhalwani NN Davies MJ Yates T Healthy lifestyle and life expectancy in people with multimorbidity in the UK Biobank: A longitudinal cohort study. PLoS Med. 2020;17:e1003332. 10.1371/journal.pmed.1003332 32960883 PMC7508366

[24] Huang X Liu J Qi L Adachi JD Wu J Li Z Birth Weight and the Risk of Cardiovascular Outcomes: A Report From the Large Population-Based UK Biobank Cohort Study. Front Cardiovasc Med. 2022;9:827491. 10.3389/fcvm.2022.827491 35402571 PMC8987713

[25] Carlsson Wallin M Ekstrom P Marsal K Kallen K Apgar score and perinatal death after one previous caesarean delivery. BJOG. 2010;117:1088–97. 10.1111/j.1471-0528.2010.02614.x 20497412

[26] Cnattingius S Johansson S Razaz N Apgar Score and Risk of Neonatal Death among Preterm Infants. N Engl J Med. 2020;383:49–57. 10.1056/NEJMoa1915075 32609981

[27] van Borkulo CD Borsboom D Epskamp S Blanken TF Boschloo L Schoevers RA A new method for constructing networks from binary data. Sci Rep. 2014;4:5918. 10.1038/srep05918 25082149 PMC4118196

[28] Chen JH Chen ZH Extended Bayesian information criteria for model selection with large model spaces. Biometrika. 2008;95:759–71. 10.1093/biomet/asn034

[29] Opsahl T Agneessens F Skvoretz J Node centrality in weighted networks: Generalizing degree and shortest paths. Soc Networks. 2010;32:245–51. 10.1016/j.socnet.2010.03.006

[30] Richetin J Preti E Costantini G De Panfilis C The centrality of affective instability and identity in Borderline Personality Disorder: Evidence from network analysis. PLoS One. 2017;12:e0186695. 10.1371/journal.pone.0186695 29040324 PMC5645155

[31] Borsboom D Robinaugh DJ Rhemtulla M Cramer AOJ Grp P Robustness and replicability of psychopathology networks. World Psychiatry. 2018;17:143–4. 10.1002/wps.20515 29856550 PMC5980315

[32] Harrell FE. Regression modeling strategies. Germany: Springer; 2001.

[33] SAS/STAT(R) 9.3 User's GuideThe MI Procedure. 2024. Available: https://support.sas.com/documentation/cdl/en/statug/63962/HTML/default/viewer.htm#statug_mi_sect001.htm. Accessed: 8 October 2023.

[34] Advanced Research Computing Statistical Methods and Data Analytics. Multiple imputation in SAS Part 1. 2023. Available: https://stats.oarc.ucla.edu/sas/seminars/multiple-imputation-in-sas/mi_new_1/. Accessed: 8 October 2023.

[35] van Borkulo CD Van Bork R Boschloo L Kossakowski JJ Tio P Schoevers RA Comparing Network Structures on Three Aspects: A Permutation Test. Psychol Methods. 2023;28:1273–85. 10.1037/met0000476 35404628

[36] Farajnezhad F Shaahmadi F Fashi Z Daaylar L Prevalence of high risk pregnancy and some relevant factors in referred women to health centers. Journal of scientific achievements. 2017;2:4–7.

[37] Kim JH Choi YY Yoo SI Kang DR Association between ambient air pollution and high-risk pregnancy: A 2015-2018 national population-based cohort study in Korea. Environ Res. 2021;197:110965. 10.1016/j.envres.2021.110965 33722528

[38] Bola R Ujoh F Lett R Identification and mitigation of high-risk pregnancy with the Community Maternal Danger Score Mobile Application in Gboko, Nigeria. PLoS One. 2022;17:e0275442. 10.1371/journal.pone.0275442 36174030 PMC9521834

[39] Jemila N Midhagsa D Melkamu G Prevalence of High Risk Pregnant Women Who Attend Antenatal Care and Associated Factors in Jimma Medical Center, Jimma Town, South Western Ethiopia. Int J Womens Health Wellness. 2021;7:133. 10.23937/2474-1353/1510133

[40] Carroll AE Why Is US Maternal Mortality Rising? JAMA. 2017;318:321. 10.1001/jama.2017.8390 28742896

[41] Liang J Mu Y Li X Tang W Wang Y Liu Z Relaxation of the one child policy and trends in caesarean section rates and birth outcomes in China between 2012 and 2016: observational study of nearly seven million health facility births. BMJ. 2018;360:k817. 10.1136/bmj.k817 29506980 PMC5836714

[42] Zhang HX Zhao YY Wang YQ Analysis of the Characteristics of Pregnancy and Delivery before and after Implementation of the Two-child Policy. Chin Med J (Engl). 2018;131:37–42. 10.4103/0366-6999.221268 29271378 PMC5754956

[43] Lumbiganon P Laopaiboon M Gülmezoglu AM Souza JP Taneepanichskul S Pang RY Method of delivery and pregnancy outcomes in Asia: the WHO global survey on maternal and perinatal health 2007-08. Lancet. 2010;375:490–9. 10.1016/S0140-6736(09)61870-5 20071021

[44] Zhu H Zhao ZJ Xu J Chen YM Zhu Q Zhou LM The prevalence of gestational diabetes mellitus before and after the implementation of the universal two-child policy in China. Frontiers in endocrinology. 2022;13:960877. 10.3389/fendo.2022.960877 36060951 PMC9433653

[45] Zelop CM Einav S Mhyre JM Martin S Cardiac arrest during pregnancy: ongoing clinical conundrum. Am J Obstet Gynecol. 2018;219:52–61. 10.1016/j.ajog.2017.12.232 29305251

[46] Carroll AE Why Is US Maternal Mortality Rising? JAMA. 2017;318:321. 10.1001/jama.2017.8390 28742896

[47] Yan J Wang L Yang Y Zhang Y Zhang H He Y The trend of caesarean birth rate changes in China after ‘universal two-child policy’ era: a population-based study in 2013-2018. BMC Med. 2020;18:249. 10.1186/s12916-020-01714-7 32928217 PMC7491061

[48] Rogers AJG Harper LM Mari G A conceptual framework for the impact of obesity on risk of cesarean delivery. Am J Obstet Gynecol. 2018;219:356–63. 10.1016/j.ajog.2018.06.006 29902446

[49] Paidas Teefey C Reforma L Koelper NC Sammel MD Srinivas SK Levine LD Risk Factors Associated With Cesarean Delivery After Induction of Labor in Women With Class III Obesity. Obstet Gynecol. 2020;135:542–9. 10.1097/AOG.0000000000003703 32028494

[50] Hu J Ye YX Lu AY Chen LW Mai YJ Huang GT Pregnancy Outcomes in Patients With Heart Disease in China. Am J Cardiol. 2020;125:1718–24. 10.1016/j.amjcard.2020.02.043 32284176

[51] Song P Liu Y Yu X Wu J Poon AN Demaio A Prevalence of epilepsy in China between 1990 and 2015: A systematic review and meta-analysis. J Glob Health. 2017;7:020706. 10.7189/jogh.07.020706 29302325 PMC5737100

[52] Ding D Zhou D Sander JW Wang WZ Li SC Hong Z Epilepsy in China: major progress in the past two decades. Lancet Neurol. 2021;20:316–26. 10.1016/S1474-4422(21)00023-5 33743240

[53] Li F Qin JB Zhang SM Chen LZ Prevalence of hypertensive disorders in pregnancy in China: A systematic review and meta-analysis. Pregnancy Hypertens. 2021;24:13–21. 10.1016/j.preghy.2021.02.001 33626437

[54] Lee SI Hanley S Vowles Z Plachcinski R Moss N Singh M The development of a core outcome set for studies of pregnant women with multimorbidity. BMC Med. 2023;21:314. 10.1186/s12916-023-03013-3 37605204 PMC10441728

[55] Zhang X Chen L Wang X Wang X Jia M Ni S Changes in maternal age and prevalence of congenital anomalies during the enactment of China’s universal two-child policy (2013-2017) in Zhejiang Province, China: An observational study. PLoS Med. 2020;17:e1003047. 10.1371/journal.pmed.1003047 32092053 PMC7039412

[56] McCauley M Zafar S van den Broek N Maternal multimorbidity during pregnancy and after childbirth in women in low- and middle-income countries: a systematic literature review. BMC Pregnancy Childbirth. 2020;20:637. 10.1186/s12884-020-03303-1 33081734 PMC7574312

[57] Gholami R Borumandnia N Kalhori E Taheri M Khodakarami N The impact of covid-19 pandemic on pregnancy outcome. BMC Pregnancy Childbirth. 2023;23:811. 10.1186/s12884-023-06098-z 37993814 PMC10664522

[58] Zhang XY Wu XB Study on Trend and Characteristics of Spatio-Temporal Evolution of COVID-19 Epidemic in China—Based on Spatial Markov Chain and STL Time Series Model. Hans Journal of Data Mining. 2022;12:8–19. Chinese. 10.12677/HJDM.2022.121002

[59] Qiao J Wang Y Li X Jiang F Zhang Y Ma J A Lancet Commission on 70 years of women’s reproductive, maternal, newborn, child, and adolescent health in China. Lancet. 2021;397:2497–536. 10.1016/S0140-6736(20)32708-2 34043953

[60] Cetin O Guzel Ozdemir P Kurdoglu Z Sahin HG Investigation of maternal psychopathological symptoms, dream anxiety and insomnia in preeclampsia. J Matern Fetal Neonatal Med. 2017;30:2510–5. 10.1080/14767058.2016.1254185 27806675

