# Supplementary material for: Burden of multiple high-risk factors in pregnancy before and after the universal two-child policy in Chinese women: An observational study
Source: J Glob Health. 2024 Jul 19;14:04134. doi: 10.7189/jogh.14.04134 (PMC11257705; doi:10.7189/jogh.14.04134)
Supplement: Online Supplementary Document [file jogh-14-04134-s001.zip › jogh-14-04134-s003.pdf]

Supplementary information

Contents

Supplementary Tables 2

Table S1. High-risk factors included in the definition of MHFP and their distributions in 2015 and 2020/2021. 2

Table S2. Basic characteristics of pregnant women in Huai'an in 2015 and 2020/2021. . 4

Table S3. Sensitivity analysis for the association of MHFP and number of risk factors with pregnancy outcomes by imputing missing covariates. 5

Table S4. Association between the first five leading combinations of risk factors and pregnancy outcomes. 7

Supplementary Figures 9

Figure S1. Flowchart of the population selection. 9

Figure S2. Network centrality measurements (strength, closeness, and betweenness) of high-risk factors in 2015 and 2020/2021. 10

Figure S3. Accuracy estimations of all edge weights using the non-parametric bootstrapping method. 11

Figure S4. Stability estimations of centrality indices using the case-drop bootstrapping method. 12

Figure S5. Subgroup analysis of the association between MHFP and pregnancy outcomes stratified by socioeconomic factors. 13

Supplementary Tables

Table S1. High-risk factors included in the definition of MHFP and their distributions in 2015 and 2020/2021.

Themes	NO.	High-risk factors in pregnancy	2015 (N=57495) n (%)	2020/2021 (N=27923) n (%)	*P* value
**Basic characteristics**	1	Maternal age ≥35 or ≤18	5747 (10.00)	3054 (10.94)	<0.001
	2	Height ≤145 cm	104 (0.18)	42 (0.15)	0.312
	3	BMI >25 or <18.5	11665 (20.29)	7531 (26.97)	<0.001
	4	Rh-negative	6 (0.01)	2 (0.01)	0.643
**Pregnancy history**	5	History of infertility	27 (0.05)	8 (0.03)	0.215
	6	History of abnormal pregnancy (including abortion ≥3 times, history of preterm birth, history of perinatal death, history of birth defect, history of ectopic pregnancy, history of trophoblastic disease, history of pregnancy complications or comorbidities)	2572 (4.47)	1971 (7.06)	<0.001
	7	Abnormal pregnancy of this pregnancy (including multiple pregnancy and assistant reproduction)	810 (1.41)	734 (2.63)	<0.001
**Diseases history in gynecology and obstetrics**	8	Reproductive malformation or contracted pelvis	258 (0.45)	149 (0.53)	0.091
	9	Uterine fibroids or ovarian cysts ≥5 cm	110 (0.19)	226 (0.81)	<0.001
	10	Scarred uterus (including history of myoma excavation, myoma excavation, uterine plastic surgery, cornual pregnancy, uterine perforation, and cesarean delivery)	9887 (17.2)	7512 (26.90)	<0.001
**Pregnancy comorbidities**	11	Cardiovascular diseases	92 (0.16)	250 (0.90)	<0.001
	12	Respiratory diseases	6 (0.01)	70 (0.25)	<0.001

	13	Digestive diseases	190 (0.33)	31 (0.11)	<0.001
	14	Urinary diseases	11 (0.02)	67 (0.24)	<0.001
	15	Hematological diseases	248 (0.43)	249 (0.89)	<0.001
	16	Endocrine diseases	280 (0.49)	1244 (4.46)	<0.001
	17	Mental disorders	20 (0.03)	54 (0.19)	<0.001
	18	Neurological diseases	151 (0.26)	28 (0.10)	<0.001
	19	Immune diseases	7 (0.01)	39 (0.14)	<0.001
	20	Cancer	0 (0.00)	4 (0.01)	0.004
**Pregnancy complications**	21	Gestational diabetes mellitus	890 (1.55)	3780 (13.54)	<0.001
	22	Hypertension disorders in pregnancy (Gestational hypertension/pre-eclampsia/eclampsia)	2885 (5.02)	1121 (4.01)	<0.001
	23	Intrahepatic cholestasis of pregnancy	97 (0.17)	154 (0.55)	<0.001
	24	Anemia during pregnancy	4343 (7.55)	2603 (9.32)	<0.001
	25	Premature rupture of membranes	5147 (8.95)	3161 (11.32)	<0.001
	26	Placenta previa	661 (1.15)	142 (0.51)	<0.001
	27	Polyhydramnios/oligohydramnios	8590 (14.94)	2607 (9.34)	<0.001
	28	Intrauterine growth restriction	127 (0.22)	150 (0.54)	<0.001
**Infectious diseases**	29	Viral hepatitis	2306 (4.01)	602 (2.16)	<0.001
	30	Tuberculosis	2 (0.00)	1 (0.00)	0.981
	31	Syphilis (+)	194 (0.34)	132 (0.47)	0.003
	32	HIV (+)	3 (0.01)	2 (0.01)	0.728
	33	Other sexually transmitted diseases (such as condyloma acuminata, gonorrhea)	27 (0.05)	21 (0.08)	0.102

Table S2. Basic characteristics of pregnant women in Huai'an in 2015 and 2020/2021.

	**Total**	**2015**	**2020/2021**	***P* value**
**Maternal age, (median (IQR))**	27.0 (6.0)	26.0 (5.0)	28.0 (6.0)	<0.001
**MHFP, n (%)**	25560 (29.9)	14833 (25.8)	10727 (38.4)	<0.001
**Hospital level, n (%)**				<0.001
Grade I	19805 (23.2)	17567 (30.6)	2238 (8.0)	
Grade II	32369 (37.9)	23028 (40.1)	9341 (33.5)	
Grade III	33244 (38.9)	16900 (29.4)	16344 (58.5)	
**Education level, n (%)**				<0.001
Middle school and below	45730 (53.5)	35419 (61.6)	10311 (36.9)	
High school or technical secondary school	18354 (21.5)	10628 (18.5)	7726 (27.7)	
Junior college and above	21250 (24.9)	11448 (19.9)	9802 (35.1)	
Missing	84 (0.1)	0 (0.0)	84 (0.3)	
**Employment status, n (%)**				<0.001
Unemployed	20363 (23.8)	19754 (34.4)	609 (2.2)	
Employed or self-employed	48336 (56.6)	33335 (58.0)	15001 (53.7)	
Others	16628 (19.5)	4406 (7.7)	12222 (43.8)	
Missing	91 (0.1)	0 (0.0)	91 (0.3)	
**BMI before pregnancy, n (%)**				<0.001
Normal weight	58270 (68.2)	40587 (70.6)	17683 (63.3)	
Underweight	6412 (7.5)	4618 (8.0)	1794 (6.4)	
Overweight	17589 (20.6)	11124 (19.3)	6465 (23.2)	
Obese	3147 (3.7)	1166 (2.0)	1981 (7.1)	
**Gravidity before this pregnancy, n (%)**				<0.001
0	32597 (38.2)	23296 (40.5)	9301 (33.3)	
1	28032 (32.8)	19938 (34.7)	8094 (29.0)	
2	13922 (16.3)	8352 (14.5)	5570 (19.9)	
≥3	10867 (12.7)	5909 (10.3)	4958 (17.8)	
**Parity before this pregnancy, n (%)**				<0.001
0	51992 (60.9)	38879 (67.6)	13113 (47.0)	
1	29542 (34.6)	17060 (29.7)	12482 (44.7)	
≥2	3884 (5.3)	1556 (2.7)	2328 (8.3)	
**Preterm birth (< 37 week)**	3841 (4.5)	2158 (3.8)	1683 (6.0)	<0.001
**Low birth weight**	2418 (2.83)	1518 (2.6)	900 (3.2)	<0.001
**High birth weight**	7963 (9.3)	5585 (9.7)	2378 (8.5)	<0.001
**Low score of 5-minute Apgar**	603 (0.7)	402 (0.7)	201 (0.7)	0.735
**Perinatal death**	451 (0.5)	287 (0.5)	164 (0.6)	0.095
**Neonatal death**	370 (0.4)	332 (0.6)	38 (0.1)	<0.001

Table S3. Sensitivity analysis for the association of MHFP and number of risk factors with pregnancy outcomes by imputing missing covariates.

Pregnancy outcomes	Total population		2015		2020/2021	
	**n (%)**	**OR (95% CI)**	**n (%)**	**OR (95% CI)**	**n (%)**	**OR (95% CI)**
**MHFP *vs.* non-MHFP**						
Preterm birth	2121 (8.3)	2.57 (2.49-2.65)	1087 (7.3)	2.75 (2.51-3.02)	1034 (9.6)	2.62 (2.50-2.75)
Low birth weight	1344 (5.3)	2.77 (2.66-2.88)	139 (5.0)	2.58 (2.32-2.88)	605 (5.6)	3.61 (3.38-3.86)
High birth weight	2614 (10.2)	1.14 (1.12-1.17)	1578 (10.6)	1.10 (1.03-1.17)	1036 (9.7)	1.24 (1.19-1.29)
Low score of 5-minute Apgar	247 (1.0)	1.41 (1.31-1.52)	154 (1.0)	1.61 (1.31-1.99)	93 (0.9)	1.36 (1.19-1.55)
Perinatal death	207 (0.8)	1.75 (1.60-1.90)	120 (0.8)	1.85 (1.45-2.36)	87 (0.8)	1.95 (1.69-2.26)
Neonatal death	164 (0.6)	1.76 (1.60-1.94)	137 (0.9)	1.80 (1.43-2.26)	27 (0.3)	4.36 (3.15-6.03)
**Number of risk factors**						
Preterm birth						
0	574 (2.1)	Ref.	341 (1.7)	Ref.	233 (3.1)	Ref.
1	1146 (3.6)	1.69 (1.61-1.77)	730 (3.3)	1.97 (1.73-2.25)	416 (4.3)	1.41 (1.31-1.52)
2	972 (5.8)	2.61 (2.49-2.74)	568 (5.4)	3.19 (2.77-3.67)	404 (6.4)	2.21 (2.05-2.39)
3	658 (10.6)	4.52 (4.28-4.77)	327 (10.1)	5.69 (4.82-6.72)	331 (11.2)	3.95 (3.64-4.28)
≥4	491 (12.8)	8.04 (7.55-8.55)	192 (18.1)	9.54 (7.73-11.78)	299 (20.0)	7.64 (7.01-8.34)
Low birth weight						
0	372 (1.3)	Ref.	271 (1.3)	Ref.	101 (1.4)	Ref.
1	702 (2.2)	1.64 (1.55-1.74)	508 (2.3)	1.74 (1.50-2.02)	194 (2.0)	1.58 (1.41-1.76)
2	632 (3.8)	2.80 (2.64-2.97)	390 (3.7)	2.79 (2.38-3.28)	242 (3.9)	3.31 (2.98-3.690
3	406 (6.6)	4.72 (4.42-5.05)	210 (6.5)	4.61 (3.80-5.59)	196 (6.6)	5.97 (5.34-6.69)
≥4	306 (12.0)	8.49 (7.79-9.16)	139 (13.1)	8.59 (6.82-10.83)	167 (11.2)	10.78 (9.55-12.16)
High birth weight						
0	2471 (8.8)	Ref.	1905 (9.3)	Ref.	566 (7.6)	Ref.
1	2878 (9.1)	1.00 (0.98-1.03)	2101 (9.5)	0.99 (0.93-1.06)	776 (8.0)	1.02 (0.97-1.07)
2	1669 (9.9)	1.10 (1.07-1.13)	1097 (10.4)	1.06 (0.98-1.15)	572 (9.1)	1.17 (1.11-1.24)
3	673 (10.9)	1.24 (1.19-1.29)	366 (11.3)	1.16 (1.03-1.31)	307 (10.4)	1.38 (1.29-1.47)
≥4	272 (10.6)	1.28 (1.20-1.36)	115 (10.8)	1.17 (0.95-1.43)	157 (10.5)	1.44 (1.32-1.58)
Low score of 5-minute						

Apgar						
0	155 (0.6)	Ref.	109 (0.5)	Ref.	46 (0.6)	Ref.
1	201 (0.6)	1.08 (0.98-1.18)	139 (0.6)	1.15 (0.89-1.48)	62 (0.6)	1.06 (0.89-1.26)
2	0.8 (0.8)	1.24 (1.12-1.39)	88 (0.8)	1.47 (1.11-1.97)	42 (0.7)	1.14 (0.94-1.38)
3	68 (1.1)	1.63 (1.42-1.86)	40 (1.2)	2.00 (1.37-2.90)	28 (1.0)	1.54 (1.24-1.92)
≥4	49 (1.9)	2.59 (2.22-3.02)	26 (2.4)	3.50 (2.23-5.49)	23 (1.5)	2.35 (1.85-2.99)
Perinatal death						
0	101 (0.4)	Ref.	67 (0.3)	Ref.	34 (0.5)	Ref.
1	143 (0.6)	1.18 (1.06-1.33)	100 (0.5)	1.34 (0.98-1.83)	43 (0.5)	1.04 (0.85-1.27)
2	106 (0.6)	1.59 (1.40-1.80)	67 (0.6)	1.82 (1.29-2.67)	39 (0.6)	1.57 (1.27-1.93)
3	64 (1.0)	2.40 (2.08-2.78)	34 (1.1)	2.72 (1.78-4.17)	30 (1.0)	2.55 (2.03-3.21)
≥4	37 (1.5)	3.07 (2.57-3.67)	19 (1.8)	3.99 (2.34-6.79)	18 (1.2)	2.97 (2.26-3.90)
Neonatal death						
0	78 (0.3)	Ref.	74 (0.4)	Ref.	4 (0.1)	Ref.
1	128 (0.4)	1.41 (1.24-1.60)	121 (0.6)	1.47 (1.09-1.96)	7 (0.1)	1.47 (0.85-2.56)
2	89 (0.5)	1.84 (1.60-2.11)	79 (0.8)	1.93 (1.40-2.67)	10 (0.2)	3.54 (2.09-6.00)
3	46 (0.7)	2.50 (2.12-2.96)	36 (1.1)	2.60 (1.73-3.92)	10 (0.3)	7.90 (4.63-13.47)
≥4	29 (1.1)	3.67 (3.00-4.48)	22 (2.1)	4.18 (2.54-6.88)	7 (0.5)	11.45 (6.44-20.38)

Table S4. Association between the first five leading combinations of risk factors and pregnancy outcomes.

	Preterm birth	Low birth weight	High birth weight	Low score of 5-minute Apgar	Perinatal death	Neonatal death
	OR (95% CI)	OR (95% CI)	OR (95% CI)	OR (95% CI)	OR (95% CI)	OR (95% CI)
**2015**						
BMI >25 or <18.5 & Scarred uterus	2.45 (1.89-3.17)	2.26 (1.67-3.07)	1.34 (1.17-1.55)	0.99 (0.54-1.81)	0.79 (0.34-7.85)	0.98 (0.47-2.05)
BMI >25 or <18.5 & Polyhydramnios/oligohydramnios	2.88 (2.14-3.87)	2.43 (1.74-3.40)	1.07 (0.90-1.39)	2.25 (1.33-3.83)	2.99 (1.64-5.45)	2.86 (1.61-5.09)
Scarred uterus & Polyhydramnios/oligohydramnios	1.29 (0.90-1.87)	1.08 (0.68-1.70)	0.66 (0.52-0.84)	1.25 (0.62-2.49)	1.74 (0.82-3.67)	1.80 (0.89-3.65)
BMI >25 or <18.5 & Maternal age ≥35 or ≤18	2.39 (1.73-3.29)	2.25 (1.55-3.28)	1.54 (1.26-1.89)	1.83 (0.97-3.45)	1.03 (0.37-2.85)	1.18 (0.47-2.95)
BMI >25 or <18.5 & Premature rupture of membranes	8.39 (6.50-10.74)	4.77 (3.43-6.61)	1.42 (1.13-1.78)	2.43 (1.26-4.68)	2.68 (1.22-5.88)	2.82 (1.30-5.68)
**2020/2021**						
BMI >25 or <18.5 & Scarred uterus	2.92 (2.36-3.62)	2.94 (2.12-4.07)	1.49 (1.27-1.75)	1.23 (0.64-2.34)	1.60 (0.79-3.28)	7.28 (1.90-27.95)
BMI >25 or <18.5 & Gestational diabetes mellitus	2.79 (2.13-3.64)	3.37 (2.31-4.94)	2.35 (1.91-2.89)	1.27 (0.59-2.71)	2.13 (1.01-4.49)	4.64 (0.84-25.70)
Scarred uterus & Maternal age ≥35 or ≤18	2.95 (2.21-3.94)	3.89 (2.61-5.82)	0.97 (0.74-1.28)	1.45 (0.62-3.36)	1.87 (0.75-4.71)	2.82 (0.29-27.36)
Scarred uterus & Anemia during pregnancy	2.66 (1.84-3.86)	2.38 (1.31-4.32)	1.09 (0.80-1.48)	0.82 (0.20-3.48)	1.28 (0.30-5.48)	10.05 (1.69-59.93)

BMI >25 or <18.5 & Polyhydramnios/oligo hydramnios	2.09 (1.44-3.05)	3.83 (2.37-3.64)	1.03 (0.72-1.48)	1.78 (0.75-4.2)	1.28 (0.39-4.20)	7.35 (1.33-40.62)

Reference group: women with no risk factor.

Models were adjuster for hospital level, education level, employment status, gravidity, and parity.

Supplementary Figures

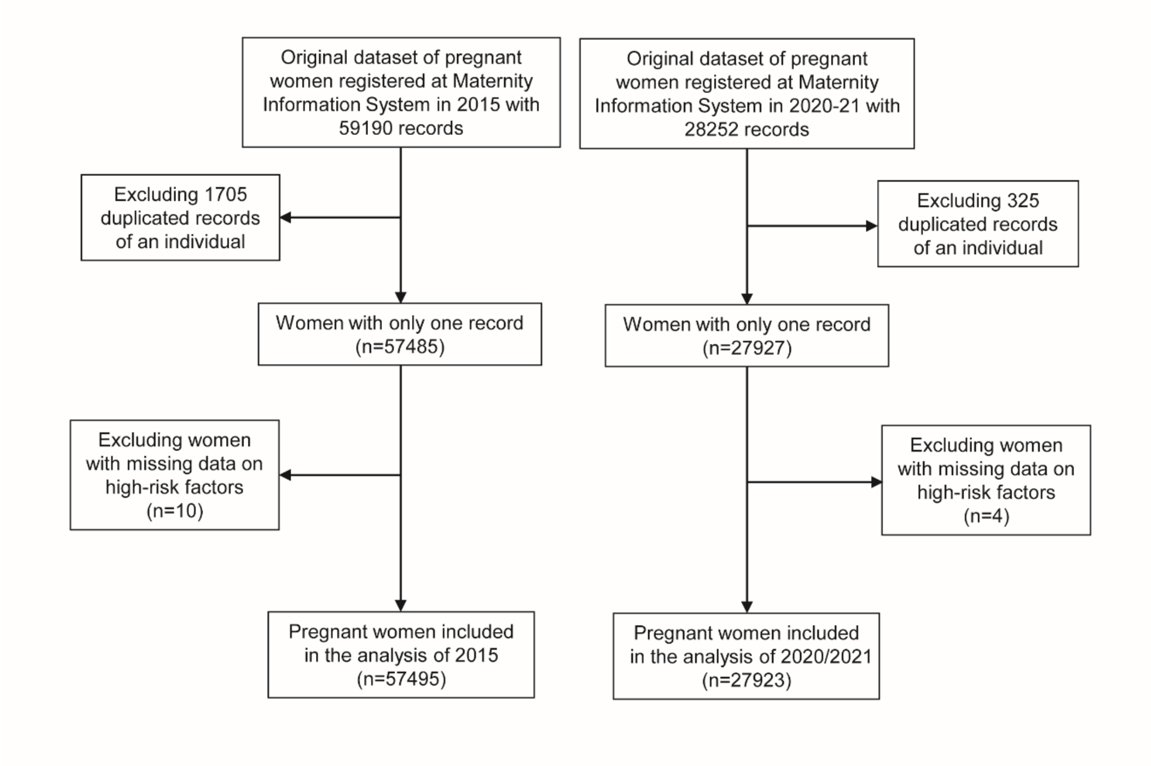

Figure S1. Flowchart of the population selection.

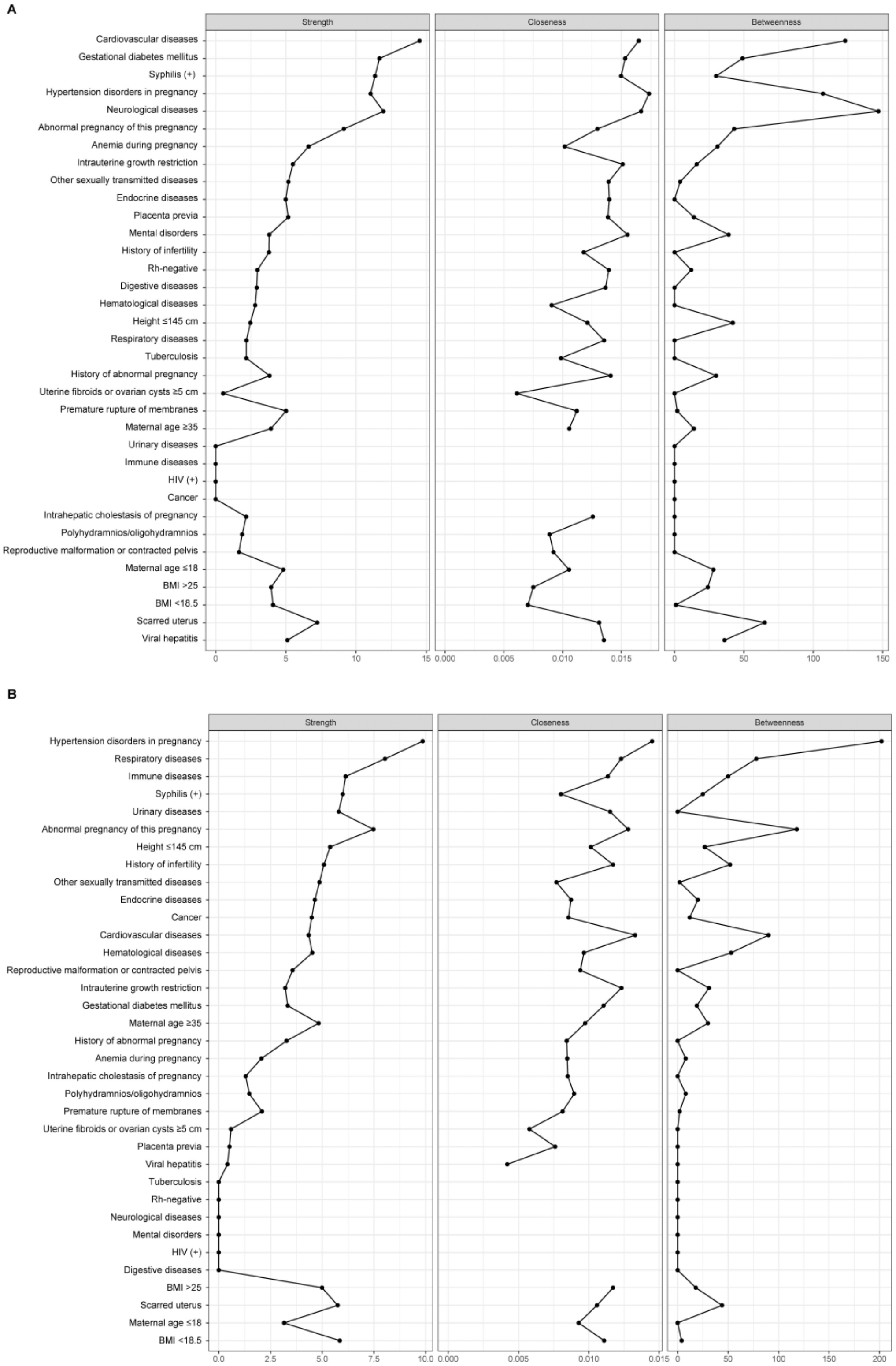

Figure S2. Network centrality measurements (strength, closeness, and betweenness) of high-risk factors in 2015 and 2020/2021.

(A) Network centrality measurements in 2015. (B) Network centrality measurements in 2020/2021.

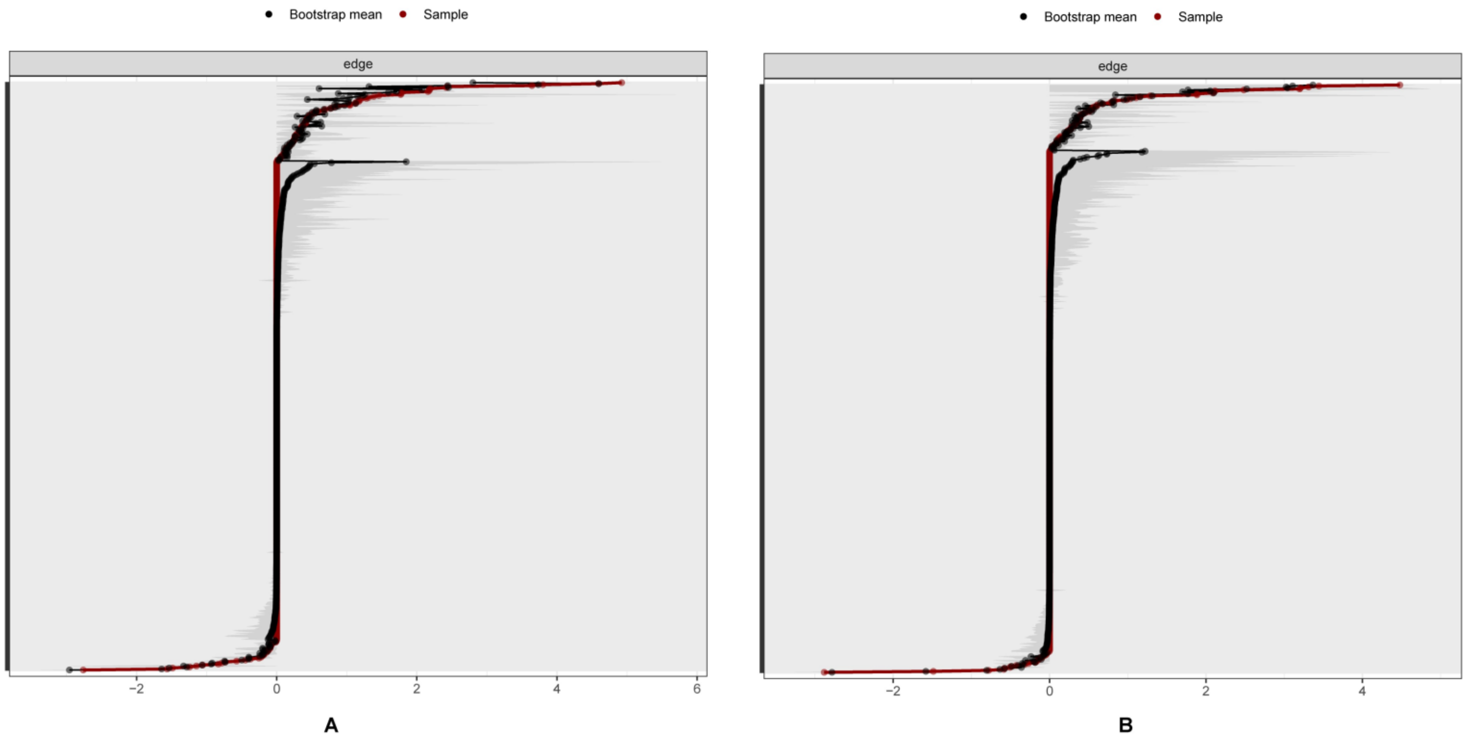

Figure S3. Accuracy estimations of all edge weights using the non-parametric bootstrapping method.

The left figure is the result of the accuracy test for 2015, the right one is that for 2020/2021. Narrower CIs indicate the reliable accuracy.

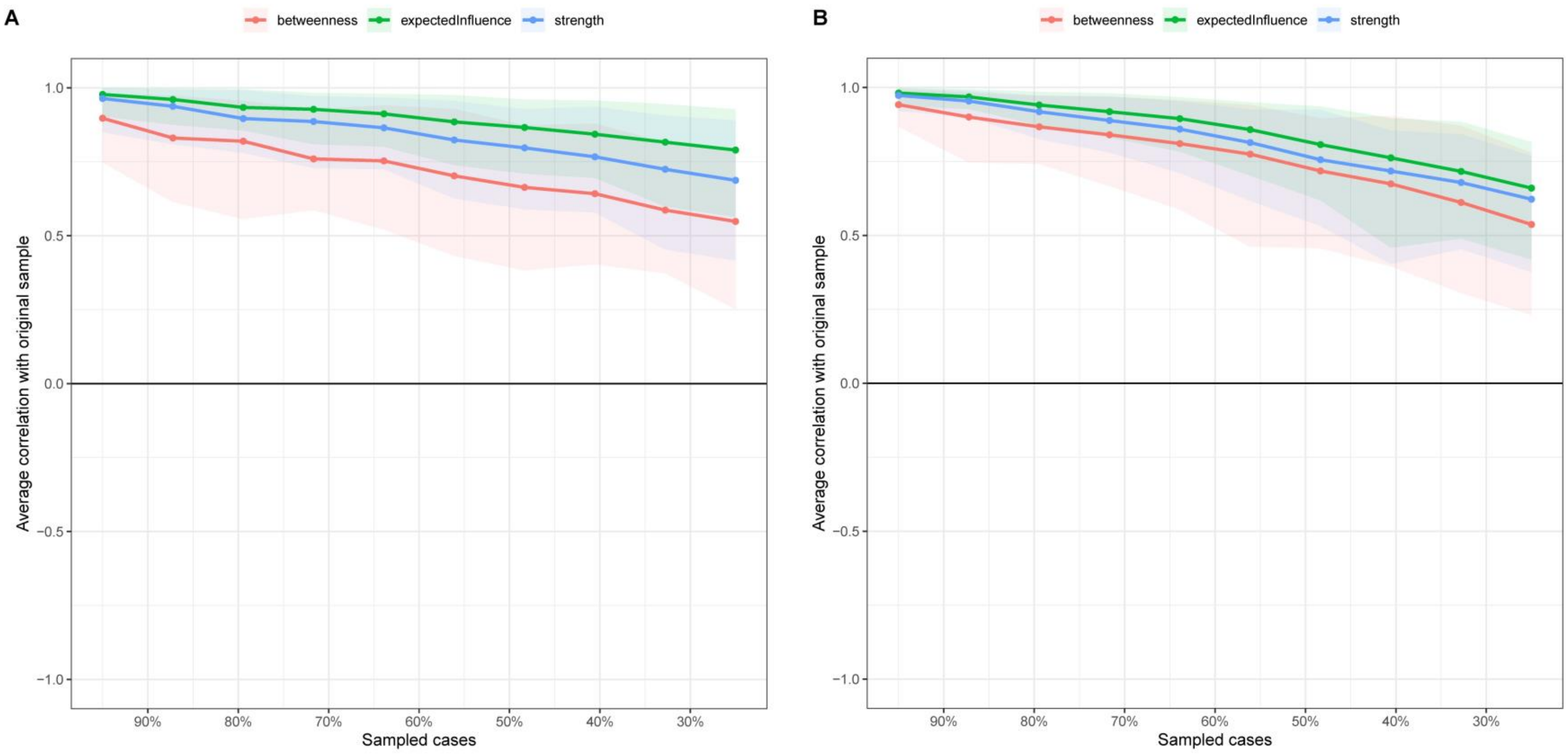

Figure S4. Stability estimations of centrality indices using the case-drop bootstrapping method.

The left figure is the result of stability test for 2015, the right one is that for 2020/2021.

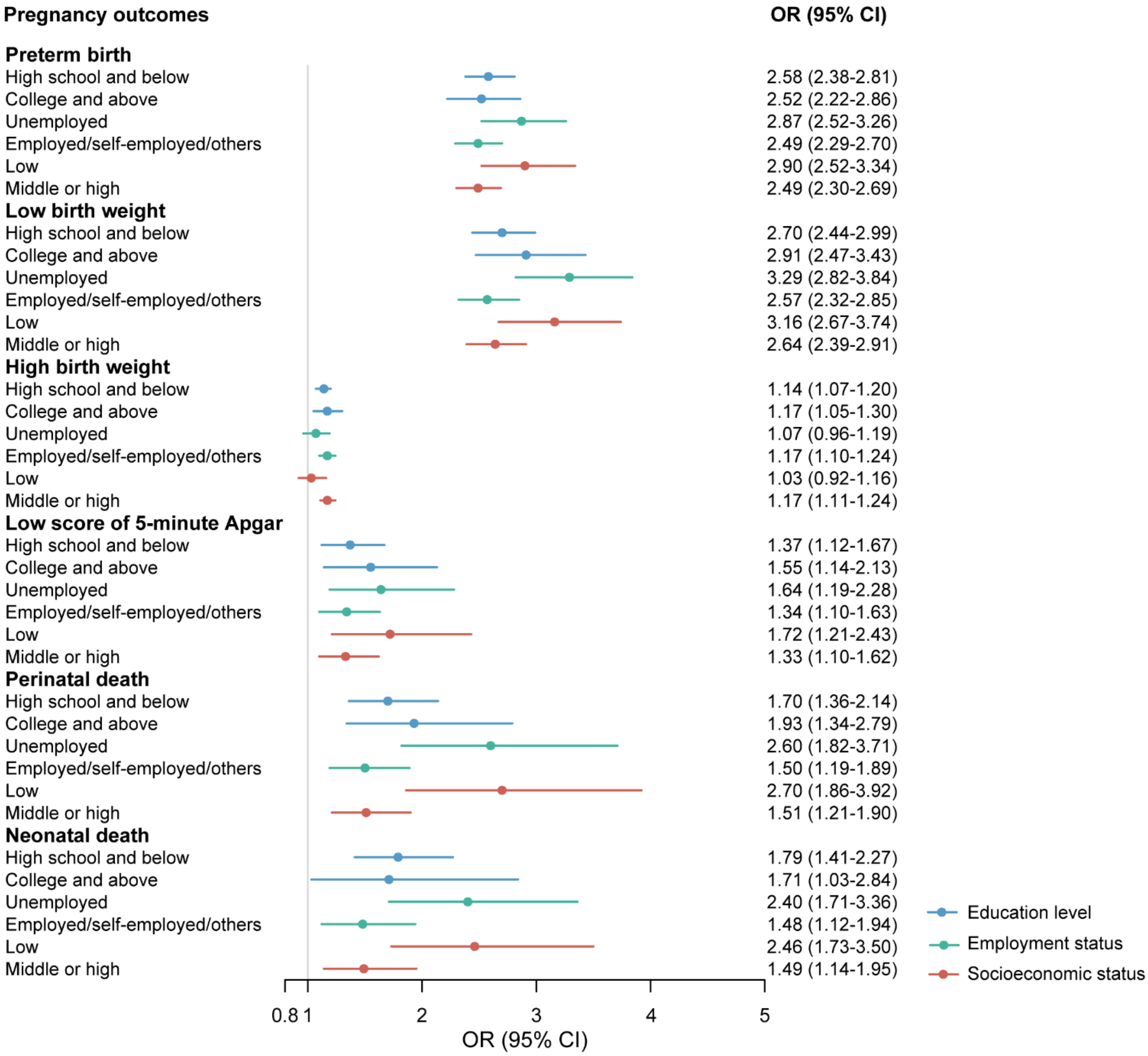

Figure S5. Subgroup analysis of the association between MHFP and pregnancy outcomes stratified by socioeconomic factors.
